# Supplementary material for: Comparative Analysis of Plant Growth-Promoting Rhizobacteria’s Effects on Alfalfa Growth at the Seedling and Flowering Stages under Salt Stress
Source: Microorganisms. 2024 Mar 19;12(3):616. doi: 10.3390/microorganisms12030616 (PMC10975677; doi:10.3390/microorganisms12030616)
Supplement: Supplementary file 1 [file microorganisms-12-00616-s001.zip › microorganisms-2901952-supplementary.pdf]

Table S1. Independent rhizobacteria and their growth-promoting traits

**Halophyte**	Strain No.	Growth promoting traits		
		Nitrogenase activity (nmolC_2H_4·h^{-1}·mL^{-1})	Phosphate solubilization ability (μg·mL^{-1})	Indole–3–acetic acid (IAA) production (μg·mL^{-1})
Tamarix chinensis	HL1	13.47	3.43	10.33
Tamarix chinensis	HL2	-	3.79	1.96
Tamarix chinensis	HL3	-	35.94	11.07
Tamarix chinensis	HL4	-	154.90	3.49
Tamarix chinensis	HL5	-	45.25	-
Tamarix chinensis	HL6	-	233.47	2.47
Tamarix chinensis	HL7	-	4.6	-
Tamarix chinensis	HL8	-	14.9	-
Tamarix chinensis	HL9	14.03	-	9.68
Tamarix chinensis	HL10	65.68	-	-
Tamarix chinensis	HL11	50.78	-	23.03

Tamarix chinensis	HL12	59.39	-	-
Tamarix chinensis	HL13	126.35	-	-
Tamarix chinensis	HL14	119.73	-	-
Tamarix chinensis	HL15	157.68	-	11.72
Tamarix chinensis	HL16	47.27	-	3.91
Tamarix chinensis	HL17	-	-	11.54
Tamarix chinensis	HL18	-	-	27.91
Tamarix chinensis	HL19	-	-	8.42
Tamarix chinensis	HL20	-	-	4.98
Tamarix chinensis	HL21	-	-	10.93
Tamarix chinensis	HL22	-	-	17.44
Tamarix chinensis	HL23	-	-	12.05
Tamarix chinensis	HL24	11.16	-	18.23
Tamarix chinensis	HL25	-	-	15.17

Tamarix chinensis	HL26	-	-	5.49
Tamarix chinensis	HL27	-	-	8.37
Tamarix chinensis	HL28	-	-	18.23
Tamarix chinensis	HL29	-	-	3.40
Tamarix chinensis	HL30	-	-	2.23
Tamarix chinensis	HL31	-	-	3.17
Tamarix chinensis	HL32	-	-	-
Lycium ruthenicum	HG1	-	-	-
Lycium ruthenicum	HG2	-	20.32	4.10
Lycium ruthenicum	HG3	117.66	-	-
Lycium ruthenicum	HG4	123.64	-	-
Lycium ruthenicum	HG5	40.09	-	0.0072
Lycium ruthenicum	HG6	75.73	-	0.89
Lycium ruthenicum	HG7	107.14	-	-

Lycium ruthenicum	HG8	210.53	3.7	21.40
Lycium ruthenicum	HG9	94.38	-	-
Lycium ruthenicum	HG10	201.69	-	2.84
Lycium ruthenicum	HG11	72.94	-	4.14
Lycium ruthenicum	HG12	289.01	2.97	-
Lycium ruthenicum	HG13	28.06	-	4.56
Lycium ruthenicum	HG14	42.88	-	5.07
Lycium ruthenicum	HG15	-	-	6.75
Lycium ruthenicum	HG16	-	-	6.42
Lycium ruthenicum	HG17	-	-	5.07
Lycium ruthenicum	HG18	-	-	7.03
Lycium ruthenicum	HG19	-	-	5.35
Lycium ruthenicum	HG20	-	-	25.17
Lycium ruthenicum	HG21	-	-	0.23

Lycium ruthenicum	HG22	-	-	10.93
Lycium ruthenicum	HG23	-	-	
Lycium ruthenicum	HG24	-	39.88	30.01
Kalidium foliatum	YZ1	38.42	-	
Kalidium foliatum	YZ2	79.63	-	
Kalidium foliatum	YZ3	90.95	-	8.79
Kalidium foliatum	YZ4	-	-	
Kalidium foliatum	YZ5	95.10	-	3.86
Kalidium foliatum	YZ6	-	-	10.33
